# Surgical management of the airway in Jarcho–Levin syndrome: a case series

**DOI:** 10.1093/jscr/rjag405

**Published:** 2026-06-22

**Authors:** Madelyn Zapata-Valentin, Patricia Mulero Soto, Luis Martinez Nater, Jan Carlos Urban, Victor Ortiz Justiniano

**Affiliations:** St. Luke's Episcopal Medical Center, Ponce, Puerto Rico; St. Luke's Episcopal Medical Center, Ponce, Puerto Rico; St. Luke's Episcopal Medical Center, Ponce, Puerto Rico; Universidad Central del Caribe, Bayamon, Puerto Rico; Ponce Health Science University, San Juan, Puerto Rico

**Keywords:** Jarcho-levin, tracheostomy, pediatric surgery, case report

## Abstract

Jarcho-Levin syndrome (JLS) is a rare genetic disorder characterized by vertebral and rib malformations, with respiratory complications being a leading cause of morbidity and mortality. Tracheostomy in patients with JLS can be technically challenging due to underlying anatomical abnormalities. We report two cases of JLS in which tracheostomy tube placement was required. In both cases, anatomical deformities presented significant technical challenges during the procedure. Based on our limited experience, we recommend specific surgical considerations for performing tracheostomy in patients with JLS. Furthermore, early tracheostomy should be considered in patients with JLS prior to the development of recurrent pneumonia and pulmonary hypertension. Currently, no standardized guidelines exist regarding the optimal timing of tracheostomy in JLS.

## Introduction

Jarcho-Levin syndrome (JLS) is a rare genetic disorder characterized by vertebral and rib malformations, short trunk dwarfism, and a constricted thoracic cage that often result in restrictive lung physiology and life-threatening respiratory insufficiency [1–4]. JLS has a notably high prevalence in Puerto Rico, where mortality rates reach up to 44% due to respiratory insufficiency secondary to pneumonia and pulmonary restriction [5–8].​

Historically, management of JLS has focused on spinal deformities and thoracic insufficiency, including spinal fusion and vertical expandable prosthetic titanium ribs, but these interventions do not directly address airway compromise [6, 9–11]. In many affected children, progressive respiratory insufficiency and recurrent infections necessitate prolonged respiratory support and intensive care [6–8]. Early tracheostomy may therefore serve as a key intervention, enabling more effective airway management, reducing pneumonia-associated morbidity, and supporting long-term respiratory stabilization, although optimal timing and technique in JLS remain undefined [4, 12, 13].​

Tracheostomy in JLS is technically challenging because of abnormal cervical anatomy, shortened neck, vertebral anomalies, and a small, malformed thoracic cage. These factors can hinder tracheal exposure and standard tube placement and may necessitate individualized surgical strategies and customized tracheostomy tubes [14–16]. This case series describes two infants with JLS and severe respiratory failure who required tracheostomy, highlighting specific technical considerations in the setting of complex anatomy and underscoring the potential role of early tracheostomy evaluation in this population.

## Case presentation

### Case 1

A male infant was born at 30 weeks of gestation to a 21-year-old G1P1 mother. Soon after birth, he was diagnosed with JLS based on characteristic vertebral and rib abnormalities on physical examination and imaging; prenatal ultrasounds had been unrevealing, and genetic testing was inconclusive. He required immediate admission to the neonatal intensive care unit (NICU) for sepsis, abdominal distension, and respiratory failure requiring mechanical ventilation.​

His postnatal course was complicated by multidrug-resistant infections, tracheitis, and disseminated intravascular coagulation. Persistent respiratory failure necessitated ongoing ventilatory support, and his markedly short neck led to repeated self-extubations and difficulty maintaining a secure airway. On transfer to our institution in critical condition, pediatric surgery was consulted for tracheostomy placement because of prolonged ventilation and recurrent extubation events. Physical examination revealed a short anterior neck with anterior deviation and limited motion. A tracheostomy was performed while the patient was on high-support synchronized intermittent mandatory ventilation.​

**Figure 1:**
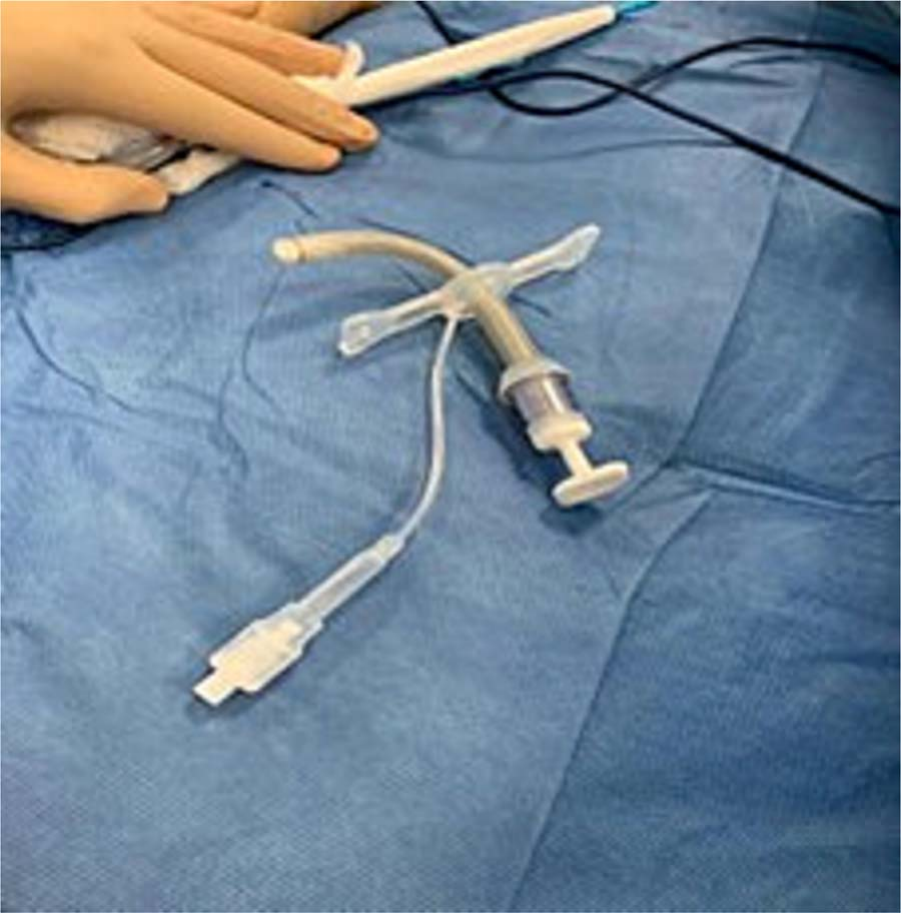
Rigid tracheostomy tube.

Despite tracheostomy, airway management remained challenging due to the short neck and close proximity of the distal trachea to the carina, which contributed to ongoing self-extubations and difficulty in maintaining optimal tube position. Imaging studies were obtained to better define the tracheal length and angulation, and a custom wire-reinforced silicone tracheostomy tube with increased rigidity and appropriate length (4.5 F × 46 mm) was designed and placed (Fig. 1). This modification allowed a more stable intratracheal position, and no intraoperative complications occurred.​

**Figure 2:**
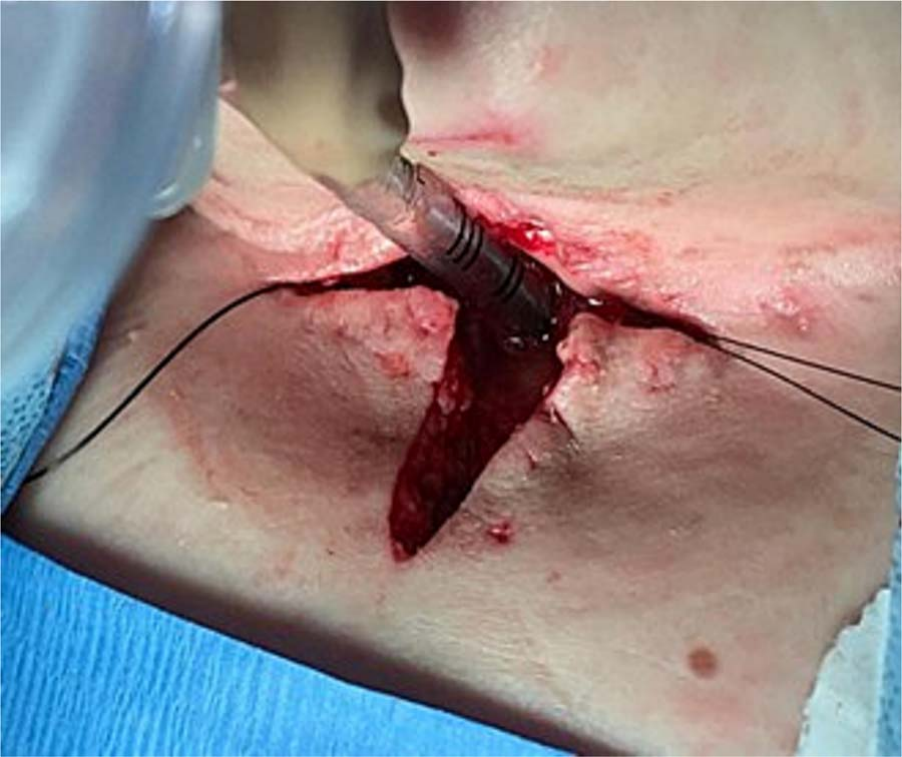
Neck incision in a T shape fashion.

**Figure 3:**
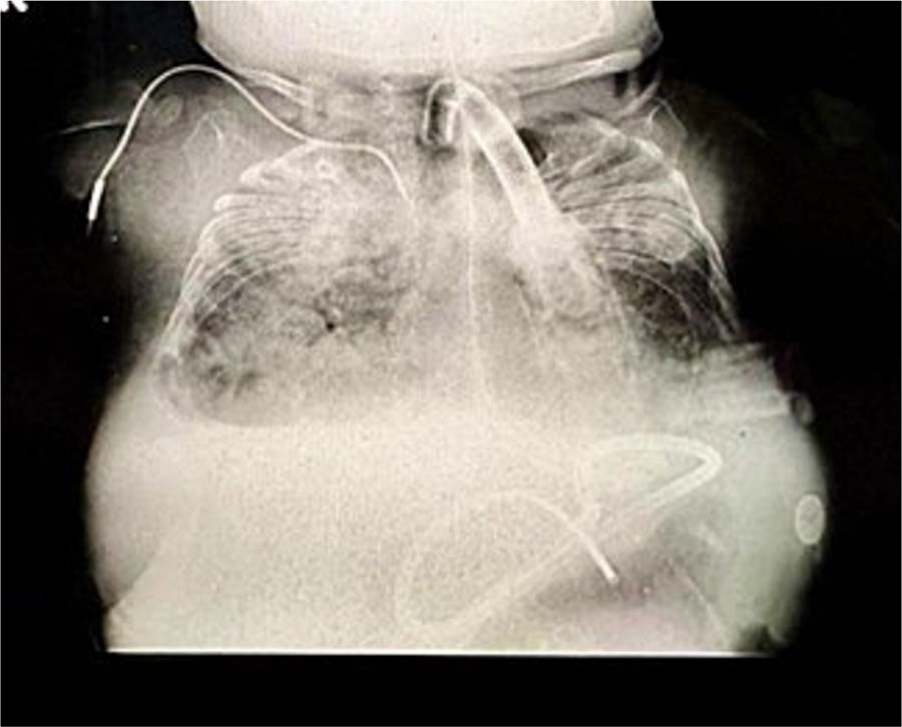
Post-operative chest X-ray.

Surgically, a transverse neck incision was made ~1 cm above the sternal notch. Dissection through the pretracheal fascia and soft tissue was limited by the short neck and restricted exposure, prompting a superior partial manubriectomy with a Lebske knife to improve access to the trachea (Fig. 2). After adequate exposure was obtained, a longitudinal tracheal incision was performed, and non-absorbable stay sutures were placed on both edges to facilitate safe manipulation during tube insertion. Proper positioning of the tracheostomy tube was confirmed with postoperative chest radiography (Fig. 3).

Following the procedure, the patient remained ventilator-dependent, and his respiratory status continued to deteriorate over subsequent months. At 13 months of age, he developed refractory pneumonia and worsening pulmonary hypertension, ultimately progressing to cardiorespiratory arrest and death despite maximal supportive care.​

### Case 2

The second patient was a female infant with a prenatal diagnosis of autosomal recessive spondylocostal dysostosis (JLS) based on a 20-week gestational ultrasound. She was born at term to a 23-year-old G2P1 mother via spontaneous vaginal delivery. Shortly after birth, she developed respiratory distress requiring nasal continuous positive airway pressure and admission to the NICU. Her early postnatal course was marked by persistent respiratory symptoms, gram-negative bacteremia, and endocarditis. Echocardiography at three months demonstrated pulmonary artery dilation and pulmonary regurgitation, and she was diagnosed with moderate pulmonary hypertension and started on sildenafil.​

During the first 6 months of life, she remained largely dependent on non-invasive ventilation and experienced an episode of respiratory failure that required brief intubation. She was eventually discharged home on non-invasive ventilatory support but continued to have recurrent respiratory decompensation. Over the following months, she was readmitted multiple times with respiratory distress requiring intubation and prolonged mechanical ventilation, with several failed extubation attempts and episodes of self-extubation attributed to her short neck anatomy. These events mirrored the airway instability seen in the first case and led to a consultation for tracheostomy placement.​

At 15 months of age, after failure to wean from mechanical ventilation during yet another admission, pediatric surgery was again consulted. Preoperative imaging was used to plan tracheostomy tube length and positioning, and a custom wire-reinforced silicone Bivona tracheostomy tube (3.5 F × 30 mm) was prepared.

**Figure 4:**
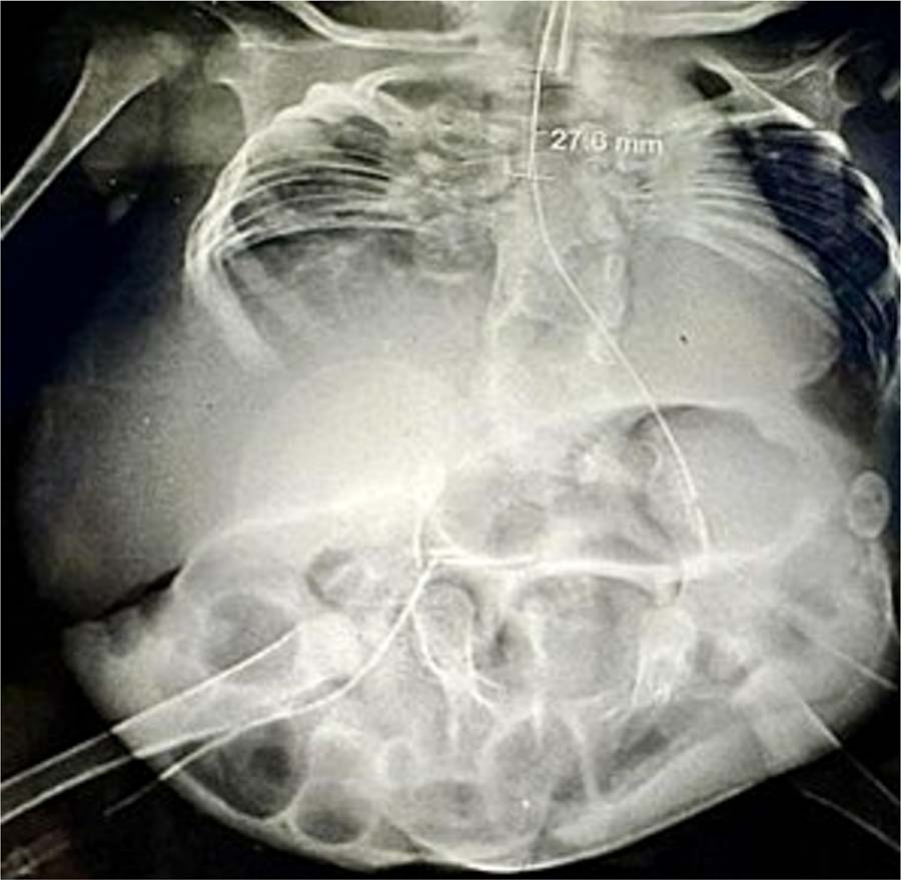
Post-operative CXR with tracheostomy tube distance from carina.

**Figure 5:**
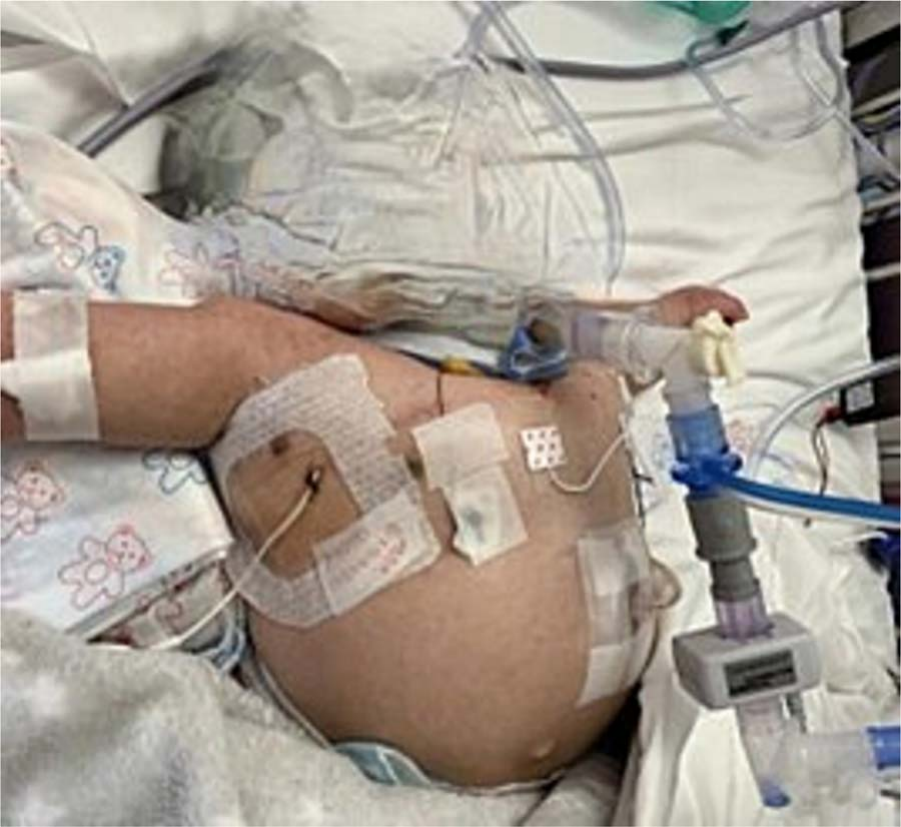
Post-operative tracheostomy tube placement on mechanical ventilation.

With the patient in a hyperextended position, a transverse incision was made ~1 cm above the sternal notch. The trachea was exposed, and the second and third tracheal rings were identified. A vertical tracheal incision was created, and the thyroid gland was divided using electrocautery to maintain hemostasis and improve access. Stay sutures were placed along the tracheal edges, and the custom cuffed tracheostomy tube was inserted without the need for manubrial resection (Fig. 4). The skin was closed in layers with nylon sutures. Postoperatively, the patient remained on synchronized intermittent mandatory ventilation with moderate ventilatory support, and there were no immediate surgical complications (Fig. 5).

Despite technically successful tracheostomy and tailored airway management, her overall clinical condition continued to worsen due to progressive pulmonary disease and pulmonary hypertension. She died 3 months later from cardiorespiratory arrest.

## Discussion

JLS encompasses a spectrum of vertebral and rib segmentation defects that produce a small, rigid thoracic cage and restrictive lung disease, predisposing affected children to recurrent respiratory infections, progressive respiratory insufficiency, and early mortality [1–4, 6–8, 11, 14, 16]. While orthopedic and thoracic interventions such as spinal fusion and vertical expandable prosthetic titanium ribs can expand thoracic volume in selected patients, they do not fully address airway instability, secretion management, or the challenges of prolonged mechanical ventilation [6, 9–11].

Published data on tracheostomy in JLS are sparse and largely limited to isolated case reports, so the true incidence and optimal timing of this procedure remain unclear [15, 16]. Tracheostomy is typically considered in pediatric patients with prolonged ventilation, difficulty with secretion clearance, severe tracheomalacia, increased airway resistance, or thoracic insufficiency unresponsive to non-invasive support, all of which may be present in JLS [12, 13, 17]. In both of our patients, recurrent respiratory failure, prolonged mechanical ventilation, and repeated self-extubations due to short neck anatomy prompted tracheostomy as a means to stabilize the airway and facilitate ongoing critical care.

Anatomical factors in JLS—including deficient tracheal cartilage, shortened cervical length, abnormal vertebral alignment, and a crowded mediastinum—make tracheostomy technically demanding. Limited neck extension and a low-lying trachea can restrict surgical exposure and increase the risk of malposition or obstruction of standard tracheostomy tubes [14–16]. Cross-sectional imaging of the neck and chest can assist with preoperative planning by delineating tracheal course, distance to the carina, and relationships to adjacent great vessels, enabling appropriate selection or customization of tracheostomy tubes [15, 16]. In our series, both patients required custom wire-reinforced silicone tubes to match their shortened tracheal length and reduce the risk of displacement; in one case, a superior partial manubriectomy was also necessary to achieve safe tracheal exposure.

Beyond technical considerations, the timing of tracheostomy in JLS is a critical but unresolved question [15, 16]. Early tracheostomy in pediatric patients requiring prolonged ventilation has been associated in some settings with improved airway stability, reduced sedation requirements, and facilitation of long-term ventilatory strategies [12, 13, 17]. In children with JLS, early tracheostomy could potentially reduce anatomic dead space, lower the work of breathing, and improve secretion clearance, with downstream effects on infection burden and pulmonary hypertension [7, 8, 12, 17]. However, the severity of underlying thoracic insufficiency and pulmonary hypoplasia may limit the extent to which airway optimization can alter overall prognosis, as illustrated by the poor outcomes in both of our patients despite technically successful procedures [6–8].

These cases suggest that early involvement of pediatric surgery and a proactive discussion of tracheostomy may be warranted in JLS patients who demonstrate recurrent respiratory failure, prolonged mechanical ventilation, or repeated extubation failures. Standardized protocols do not yet exist, but a multidisciplinary approach that includes neonatology, pediatric intensive care, pulmonology, cardiology, anesthesia, and surgery is essential for weighing the risks and benefits of early tracheostomy on an individual basis [13, 15–17].

Our report is limited by its retrospective nature, the small number of patients, and the absence of detailed quantitative respiratory data before and after tracheostomy. Genetic mutation data were not available, precluding genotype–phenotype correlation. Nonetheless, the cases provide practical insight into surgical strategy and airway planning in JLS and highlight the need for prospective, multi-institutional studies to better define indications, timing, and outcomes of tracheostomy in this high-risk population.​

## Conclusion

Jarcho-Levin syndrome is a rare congenital disorder with high respiratory morbidity and mortality driven by thoracic insufficiency, restrictive lung disease, and recurrent infections [1–3, 6–8]. The complex cervical and thoracic anatomy in these patients makes tracheostomy technically challenging and requires careful preoperative imaging, individualized surgical planning, and, in some cases, customized tracheostomy tubes and chest wall modification.​

This case series illustrates that tracheostomy can be performed safely in infants with JLS using tailored surgical approaches, including partial manubriectomy when needed to achieve adequate tracheal exposure. Early evaluation for tracheostomy should be considered in JLS patients who require prolonged mechanical ventilation or experience recurrent extubation failure, with the goal of optimizing airway management and potentially mitigating respiratory complications, even though prognosis may remain guarded due to underlying disease severity. Further research and multi-center collaboration are needed to develop evidence-based guidelines for timing and technique in this complex patient population.
